# High-Performance Low-Voltage Transparent Metal-Semiconductor-Metal Ultraviolet Photodetectors Based on Ultrathin Gold Asymmetric Interdigitated Electrodes

**DOI:** 10.3390/mi14071447

**Published:** 2023-07-19

**Authors:** Jianfeng Huang, Liu Yang, Sailing He

**Affiliations:** 1 Centre for Optical and Electromagnetic Research, National Engineering Research Center for Optical Instruments, College of Optical Science and Engineering, Zhejiang University, 866 Yuhangtang Road, Hangzhou 310058, China; 2 Ningbo Research Institute, Zhejiang University, Ningbo 315100, China; 3 Joint Research Center of Photonics, School of Electrical Engineering, Royal Institute of Technology (KTH), S-100 44 Stockholm, Sweden

**Keywords:** transparent ultraviolet photodetector, ultrathin metal film, asymmetric interdigitated electrodes

## Abstract

A high-performance, low-voltage, transparent, metal-semiconductor-metal ultraviolet (UV) photodetector (PD) is proposed and experimentally demonstrated, based on gold (Au) asymmetric interdigitated (aIDT) electrodes with thicknesses well below 10 nm. A 7-nm-thick Au film, with a visible transmittance of 80.4% and a sheet resistance of 11.55 Ω/sq, is patterned into aIDT electrodes on a ZnO active layer, whose average visible transmittance is up to 74.3%. Meshing the pads further improves the overall transmittance of the device. Among all fabricated devices, the PD with the aIDT finger width ratio of 1:4 performs the best. Very low dark currents are achieved at 0, 0.5 and 1 V, allowing for high responsivities and specific detectivities to the UV light. It is also a fast device, especially under the biases of 0.5 and 1 V. The comprehensive performances are comparable and even superior to those of the reported devices. The asymmetric Schottky junctions induced by the aIDT electrodes under UV illumination are the main mechanism for the low-voltage operation of our transparent PD, which is promising to be applied widely.

## 1. Introduction

Ultraviolet (UV) photodetectors (PDs) are devices which convert UV signals into electricity, which have a wide range of applications, e.g., substance analysis, flame detection, UV communication, astronomical research, etc. [1,2]. Because of the see-through feature, transparent UV PDs have additional applications in emerging transparent functional devices, such as secure optical communication [3] and smart windows [4], and therefore have attracted much attention [5,6,7].

A transparent UV PD requires both the semiconductor active layer and the electrodes to be transparent in the visible regime. In this wavelength range, wide-bandgap semiconductors, e.g., ZnO, TiO_2_, and GaN, are non-absorptive and therefore have been widely applied as the active layer [5,6,7]. Two-dimensional perovskites have also attracted attention for their ultrathin thickness, but the synthesis process is complicated [8]. In most PDs, the active layer is sandwiched between two transparent conducting electrodes (TCEs), e.g., indium tin oxide (ITO) [9,10,11,12], fluorine-doped tin oxide (FTO) [3,4,13,14], Ag nanowires (NWs) [3,14,15,16], and Ag oxide [10]. They are optically not good because of the double TCE layers limiting the visible transparency. In comparison, an in-plane metal-semiconductor-metal (MSM) configuration is favorable for a higher transparency, where TCEs are on the same surface of the semiconductor layer [17,18,19,20,21,22,23,24]. The opposite electrodes can be far apart from each other and their visible transparency is often neglected [17,18,19]. The large distance leads to a longer time for photocarriers to transport from one electrode to another and thus a slow response of the PD. Interdigitated (IDT) fingers introduced between the opposite electrodes are more advantageous and can significantly improve the transportation and collection of photocarriers [20,21,22,23]. In this case, the electrodes must be made highly transparent, e.g., using ITO [21,22] and ultrathin Ag film [23], to make the whole device appear visibly blind. Nevertheless, there are still reports that do not consider the transparency of the IDT electrodes [20,21].

On the other hand, power consumption is another important issue to be addressed by a transparent UV PD. Photoconductor-based PDs usually need high operation voltages to drive photocarriers to the outer circuit because of the lack of any inner forces [19,20,21,23]. To reduce the applied voltage, electrically symmetric Schottky junctions have been reported in a vertically configured UV PD based on Ag NWs/ZnO NWs/Ag NWs [15]. They have also been reported in in-plane MSM configurations with, e.g., symmetric FTO IDT electrodes on TiO_2_ [4] and symmetric ITO IDT electrodes on GaN [22]. With the assistance of the internal built-in potential of the junctions, photocarriers can be easily separated and driven quickly to the outer circuit, forming a photocurrent under low biases. Self-powering performance can even be achieved with electrically asymmetric heterojunctions based on, e.g., AgNWs/TiO_2_/FTO [3], Au/NiO/ZnO/ITO [11], ITO/Ga_2_O_3_/ZnO/ITO [12], Ag NWs/NiO/TiO_2_/FTO [14] and SnO_2_/NiO [25]. However, electrically asymmetric junctions have never been reported in highly transparent in-plane MSM configured UV PDs.

Au asymmetric IDT (aIDT) electrodes have been proven to enable UV detection under zero bias by forming asymmetric Schottky junctions on ZnO and β-Ga_2_O_3_ [26,27]. However, the Au aIDT electrodes are rather thick and block the transmission of visible light. Thus the PDs are opaque. The shadowing effect also leads to a poor UV response. In our previous work [23], the shadow issue was successfully addressed by sputtering sub-10 nm thick Ag IDT electrodes on ZnO, which served as both the active layer of the transparent PD and the seed layer to suppress the Volmer–Weber mode in the initial stage of physical vapor deposition [28,29]. Due to the identical Ohmic contacts formed at the Ag/ZnO interfaces, no rectifying effect was observed, and the PD needed large biases to work.

Based on the above analysis, in this paper, we propose and experimentally present a high-performance low-voltage visible-blind MSM UV PD based on electrically asymmetric Schottky junctions formed by patterning a sub-10 nm thick Au film into aIDT electrodes on top of a ZnO active layer. The ZnO also assists ultrathin Au film deposition. The pads are further gridded to improve the transparency of the whole device. With the ultrathin Au aIDT electrodes, our PD is highly transparent and achieves very good comprehensive performances at 0, 0.5 and 1 V, which are comparable and even superior to those of the previously reported transparent UV PDs working at the same voltages [3,4,10,11,14,15,17,18,22]. The low-voltage working mechanism of the PD is systematically investigated. The fabrication process is simple and scalable, endowing our transparent UV PD with a great promise of wide applications.

## 2. Materials and Methods

### 2.1. Fabrication of Ultrathin Au Films

Pieces of silica glass (25 mm × 25 mm × 0.5 mm, JGS2, RDMicro, Suzhou, China) were chosen as the transparent substrates for the deposition of Au films with thicknesses of no more than 20 nm via a magnetron sputter (Kurt J. Lesker PVD75, Jefferson Hills, PA, USA). Before sputtering, the silica substrates were ultrasonically cleaned with acetone and isopropanol in sequence, each for 5 min, and then dried with nitrogen. To suppress the Volmer–Weber growth mode during the initial stage of the film growth via sputtering, a ~25 nm thick ZnO film was introduced as a seed layer and sputtered at a rate of 0.4 Å s^−1^ (RF power: 100 W, argon pressure: 3 mTorr, room temperature). Without opening the chamber, a Au film was deposited at a rate of 4.1 Å s^−1^ (DC power: 100 W, argon pressure: 3 mTorr, room temperature). The film deposition rate was obtained by linearly fitting the different film thicknesses measured with a surface profiler (Dektak 150, Veeco, Plainview, NY, USA) versus the different sputtering duration. The film thickness could be tuned by changing the deposition time. With the ZnO seed layer, a uniform and continuous Au film could be obtained with a thickness of less than 10 nm (to be demonstrated in Section 3.1). For comparison, Au films with different thicknesses were directly sputtered onto the silica substrates without the ZnO seed layer.

### 2.2. Fabrication of UV PDs

Our UV PD consists of a ~100 nm thick ZnO active layer and ultrathin Au aIDT electrodes. As mentioned above, the ZnO active layer also serves as the seed layer for the ultrathin Au film growth. For fabrication, the ZnO was first sputtered onto a piece of clean silica substrate, on top of which a ~1.2 μm thick photoresist (PR; AZ5214, Merck, Darmstadt, Germany) was spin-coated. After UV light exposure and development, the aIDT pattern from the photomask was transferred to the PR film. Then, the thin Au film was sputtered onto the patterned PR and subsequently lifted off in acetone ultrasonically. The Au film deposited on top of the PR was removed, while the film directly deposited on the ZnO layer remained as the aIDT. All the sputtering parameters were the same as described above.

### 2.3. Characterization

Surface morphologies of the ultrathin Au films were inspected via a scanning electron microscope (SEM; Ultra 55, Carl Zeiss, Jena, Germany). To avoid charging, a piece of conductive carbon tape was attached to the film, which was electrically connected to the metallic sample holder underneath. In this case, the unattached part of the Au film could be seen clearly with the electrons. Transmittance spectra of the Au films and the UV PDs were measured via a home-built spectrophotometer based on an integrating sphere. A home-built, four-point probe system was used to measure the sheet resistances of the Au films. X-ray diffractometer (D8 ADVACNCE, Bruker, Billerica, MA, USA) was employed to analyze the crystallinity of the ZnO active layer. Optical microscope was used to observe the microstructure of the PDs. The current-voltage characteristic curves of the PDs were measured with a source meter (2450, Keithley, Cleveland, OH, USA) either in darkness or under light illumination at 365 nm from a UV LED. The light intensity was 8.62 mW/cm^2^. Transient responses of the PDs were characterized through switching the UV LED on and off manually or with programmable linear DC power supply (DP711, RIGOL, Suzhou, China).

## 3. Results and Discussion

### 3.1. Continuous Sub-10 nm Thick Au Film with High Transparency and High Conductivity

**Figure 1:**
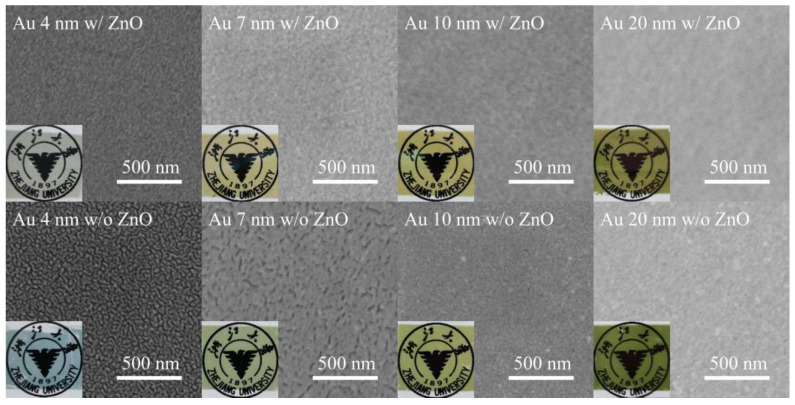
SEM images of the Au films with different thicknesses sputtered on silica substrates with and without 25 nm thick ZnO seed layers. Inset of each panel: photo of the sample with the corresponding thickness.

Figure 1 shows SEM images of the Au films with different thicknesses sputtered on top of the silica substrates with and without a 25 nm thick ZnO seed layer. The 4 nm thick film deposited on the bare silica substrate is obviously not continuous and isolated Au nanoparticles can clearly be seen. As thickness increases to 7 nm, the isolated nanoparticles become larger and coalesce. The film finally becomes continuous at larger thicknesses of 10 and 20 nm. In contrast, the film deposited on the ZnO coated silica substrate is obviously smoother than the counterpart on the bare silica substrate, even at a thickness of 4 nm. The Au film becomes continuous at a thinner thickness, i.e., 7 nm, earlier than the film sputtered directly on the bare silica substrate. This is consistent with the results of previous experiments [29,30], and is mainly attributed to the good wettability of ZnO for Au deposition, which reduces the percolation threshold thickness of the Au film [28].

Because of the smoother surfaces, the ZnO-seeded Au film looks more transparent than those without the seed layer at the same thickness and the bottom university logo can be seen more clearly (inset picture of each panel of Figure 1). We can also see from these inset pictures that the Au films on the bare silica substrate are easily damaged by the carbon tape used for the SEM inspection, meaning poor attachment to the substrate. In comparison, the ZnO-seeded Au films are more strongly attached to the silica substrate and no apparent damage can be seen, even for the 4 nm thick film.

**Figure 2:**
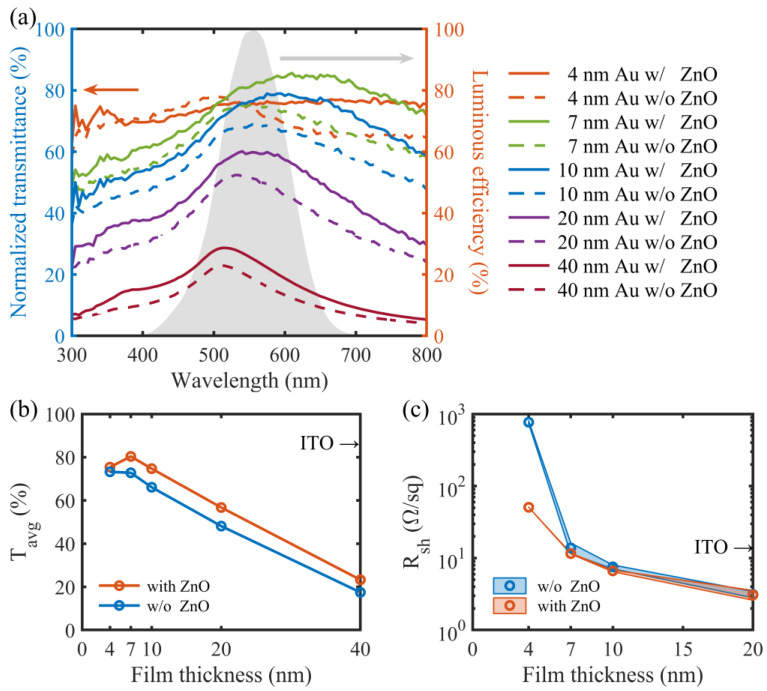
(**a**) Normalized transmittance spectra of Au films to the transmittance spectra of the silica substrate and the 25 nm thick, ZnO-coated silica substrate. The CIE 10-deg luminous efficiency function is indicated by the light gray area and characterized by the right axis. (**b**) Average transmittance, *T_avg_*, and (**c**) sheet resistances, R_sh_s, of Au films in comparison with those of ITO [31]. To obtain the data in (**c**), for each kind of film at a certain thickness, two samples were fabricated. For each sample, R_sh_s at two sites were measured and their average value at a given film thickness was plotted. The area between the two R_sh_ curves was indicated as a shadow, representing the fabrication repeatability. The average value of the four R_sh_s of the two samples either with or without the ZnO seed layer was also plotted and indicated as a circle.

To quantitatively demonstrate the optical transparency of the Au film samples, we measured their transmittance spectra and normalized them to the spectrum of the silica substrate either with or without the ZnO seed layer. Figure 2a shows the normalized transmittance spectra, *T*(*λ*), of the Au films with thicknesses ranging from 4 to 40 nm. The average transmittance, *T_avg_*, of each sample was obtained by weighting *T*(*λ*) with the CIE 10-deg luminous efficiency function, *V*(*λ*) (Figure 2a) using Equation (1), and demonstrated in Figure 2b.

(1) $$T_{avg}=\frac{\int T(\lambda )V(\lambda )d\lambda }{\int V(\lambda )d\lambda }$$

At 4 nm, the Au film on the bare silica substrate consists of isolated Au nanoparticles (Figure 1), where localized surface plasmon resonances (LSPRs) can be excited and lead to strong light scattering and absorption and thus, an obvious transmittance dip (Figure 2a) [28,29]. Due to the smaller Au nanoparticles in the 4 nm thick ZnO-seeded Au film, no apparent dip exists in the transmittance spectrum, which however is still not very high over the whole wavelength range. At 7 nm, the ZnO-seeded Au film becomes continuous (Figure 1) and its transmittance spectrum is enhanced, becoming much higher than that of the incomplete Au film on the bare substrate, especially in the long wavelength range from 500 to 800 nm (Figure 2a). A peak of around 500–600 nm is observed in the transmittance spectrum, which is consistent with previous results [28]. For both cases with and without the ZnO seed layer, further increasing the thickness of the absorptive Au film degrades the transmittance spectrum (Figure 2a) with *T_avg_* decreasing almost linearly (Figure 2b) due to the increased light absorption and reflection. Therefore, *T_avg_*’s peak is at 7 nm. It is 80.4% and 72.8% for the Au films deposited with and without the ZnO seed layer, respectively. The former is only slightly inferior to ITO [31] in terms of optical transparency (Figure 2b).

On the other hand, the sheet resistances, R_sh_s, of the Au films both with and without the ZnO seed layer were characterized. For each kind of film at a certain thickness, two samples were fabricated with identical processes. For each sample, the R_sh_s of two sites were measured and their average values at a given film thickness were plotted in Figure 2c. Here, the average value of the four R_sh_s of two samples either with or without the ZnO seed layer was also plotted a function of the film thickness. It can clearly be seen that the fabrication process and the measurement are highly repeatable and the films are quite uniform, especially for the films with thicknesses of less than 20 nm on the ZnO seed layers. R_sh_s of the Au films both with and without the ZnO seed layer decrease first quickly when the film thicknesses increase from 4 to 7 nm and then slowly when the film thicknesses are beyond 7 nm. This is mainly attributed to the morphological transition from discontinuity to continuity (Figure 1) and the increasing charge transport paths. Note that the ZnO-seeded Au films are always more conductive, with smaller R_sh_s than the unseeded Au films, when the film thicknesses are less than 20 nm. This again confirms that the smoother and more complete ultrathin film are obtained with the ZnO seed layer for wetting. The R_sh_ difference of the two cases decreases with the increasing film thickness and the R_sh_s are almost equal to each other at 20 nm. At 7 nm, the seeded Au film has a R_sh_ value as low as 11.6 Ω/sq—more conductive than both the unseeded Au film (13.8 Ω/sq) and ITO (13.36 Ω/sq) [31]. In general, the 7 nm thick seeded Au film is neither too thin electrically nor too thick optically, and is therefore quite suited to serve as electrodes of transparent UV PDs. Although the 4 nm thick Au film, either with or without the ZnO seed layer, shows the best transparency in the wavelength range of 300–500 nm among all films, it is probably not a good choice to form electrodes of a transparent UV PD, compared to the 7 nm thick Au film. This is not only because of its lower visible transparency but also due to the much higher sheet resistance, even for the film on the ZnO seed layer (50.7 Ω/sq).

### 3.2. High-Performance, Low-Voltage, Transparent MSM UV PDs Based on Asymmetric Sub-10 nm Thick Au Interdigital Electrodes

**Figure 3:**
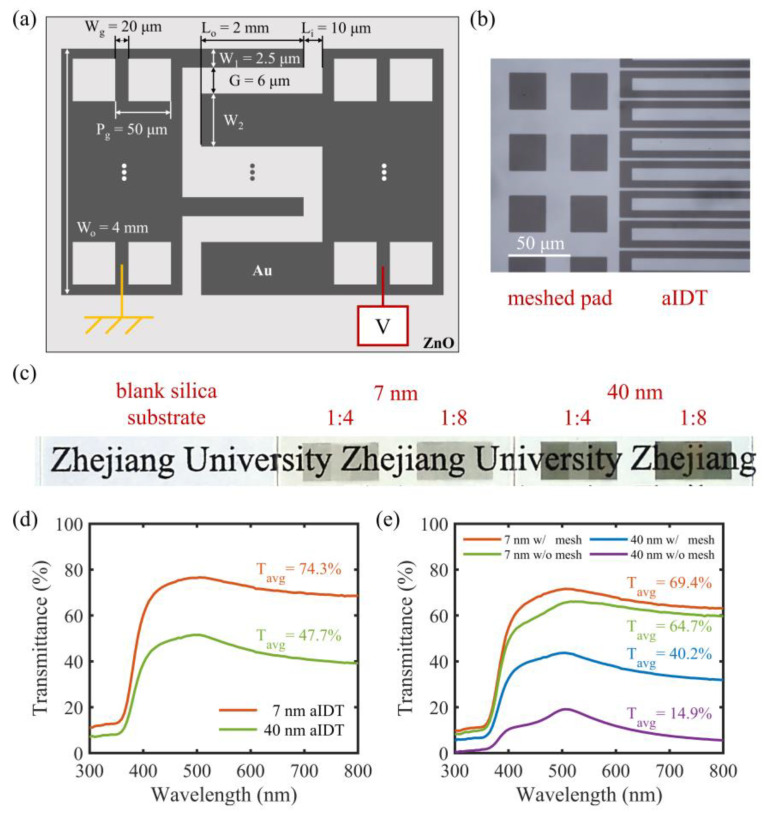
(**a**) Schematic diagrams of the aIDT electrode designs of our transparent UV PDs, where the pad with narrow fingers is grounded and the other pad with wide fingers is biased. (**b**) Microscopic image of the 1:4 aIDT and meshed pad area of the transparent UV PDs with 7 nm thick electrodes. (**c**) Photos of a blank silica substrate, transparent UV PDs with 7 nm thick 1:4 and 1:8 aIDT electrodes, as well as opaque UV PDs with 40 nm thick 1:4 and 1:8 aIDT electrodes. Measured transmittance spectra for different areas of the devices: (**d**) the 1:4 aIDT areas and (**e**) the pad areas with (w/) and without (w/o) meshes with the Au film thicknesses of 7 and 40 nm.

Based on the 7 nm thick Au film, we designed a pair of asymmetric interdigital (aIDT) electrodes on a 100 nm thick ZnO layer to form a transparent MSM UV PD operating under a low voltage. As shown in Figure 3a, the asymmetric ratio between the finger widths, i.e., W_1_:W_2_, was varied from 1:1 to 1:32 by changing W_2_ while keeping W_1_ = 2.5 μm to study the influence of asymmetric ratio on the PDs. To further increase the optical transparency of the device, the solid Au films in the pad areas were patterned into periodic square meshes. The grid line width is W_g_ = 20 μm and period is P_g_ = 50 μm. The overlapping area of the aIDT electrodes is 2 mm × 4 mm and the finger spacing is G = 6 μm. All the basic geometric parameters were indicated in Figure 3a. The microscopic image in Figure 3b demonstrates a well-patterned 7 nm thick mesh in the pad area and well-separated aIDT electrode fingers, which can effectively avoid shortage between them. Figure 3c demonstrates the fabricated transparent UV PDs with 7 nm thick 1:4 and 1:8 aIDT electrodes as examples. The meshed pads are almost as transparent as the aIDT area. For comparison, opaque UV PDs with 40 nm thick 1:4 and 1:8 aIDT electrodes are also prepared and look much darker than the corresponding transparent PDs based on the more transparent 7 nm thick electrodes.

The transmittance spectra of the 1:4 aIDT areas of the transparent and opaque UV PDs were measured and plotted in Figure 3d and Figure 3e, respectively. Compared with the 40 nm thick aIDT electrodes, the 7 nm thick aIDT electrodes are much more transparent, especially in the visible regime. Its *T_avg_* is up to 74.3% (versus 47.7% for the thick aIDT electrodes). Even in the UV regime, its transmittance is also a little bit higher (Figure 3d). Such a high UV-visible transmittance in the aIDT area is mainly attributed to the high UV-visible transmittance of the 7 nm thick Au film (Figure 2a,b). This is beneficial not only for the visual transparency but also for the UV light absorption and thus the improvement of the responsivity of the PD. In the pad areas (Figure 3e), the 7 nm thick Au film is also more advantageous optically (*T_avg_* = 64.7%) than the 40 nm thick Au film (*T_avg_* = 14.9%) and the meshing helps further improve the optical transparency (*T_avg_* = 69.4% and 40.2% for the 7 and 40 nm thick Au films, respectively).

**Figure 4:**
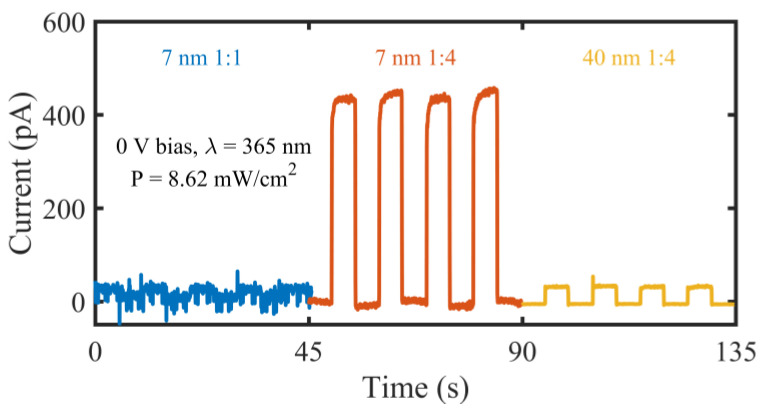
Transient currents of the transparent UV PDs with 1:1 and 1:4 7 nm thick aIDT electrodes under zero bias when the UV LED (*λ* = 365 nm, P = 8.62 mW/cm^2^) is switched on and off periodically. Transient currents of the opaque 1:4 UV PDs (whose aIDT electrode thicknesses are 40 nm) are also plotted for comparison.

Under zero bias, the transparent UV PDs with 1:1 and 1:4 aIDT electrodes were characterized when the UV LED was switched on and off periodically. Their transient output currents were plotted in Figure 4, where the transient current of the opaque 1:4 PD was also plotted for comparison. For the 1:1 transparent device, the photocurrent nearly cannot be distinguished from the dark current and noise, meaning rather weak responses to the UV light; while the 1:4 transparent device shows much more significant responses with much higher and more stable photocurrents upon lighting. The opaque 1:4 PD also shows responses to the UV light, though the responses are much weaker than those of the transparent counterpart. These phenomena clearly demonstrate the important role of the asymmetry of the IDT electrodes in the self-powered performance. The high UV transmittance of the 7 nm thick Au film (Figure 2a,b) contributes to the enhanced light absorption and the generation of photocarriers, leading to the photoresponse of the transparent PD surpassing that of the opaque one. This is similar to the behavior of the 7 nm thick Ag film reported in our previous work [23].

**Figure 5:**
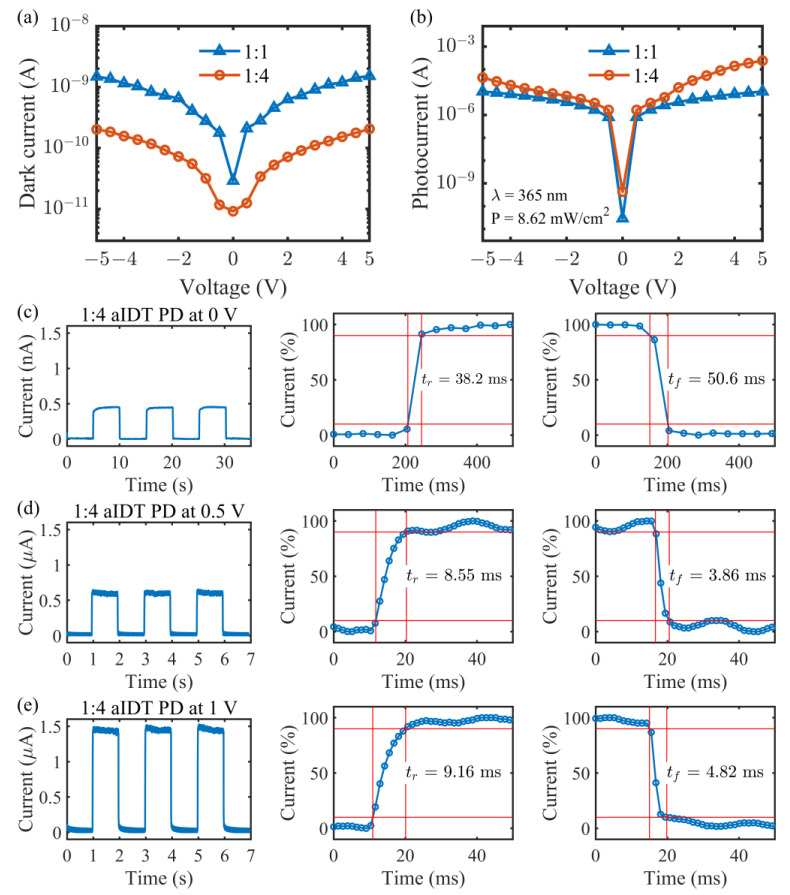
I-V characteristics (**a**) in darkness, and (**b**) under UV illumination (365 nm; ~8.62 mW/cm^2^). (**c**–**e**) Transient responses for the transparent 1:4 aIDT PD at (**c**) 0 V, (**d**) 0.5 V, and (**e**) 1 V. The rise (fall) time is defined as the time needed for the current to go from 10% (90%) to 90% (10%) of the maximum.

To further investigate the effect of the aIDT electrodes, I-V characteristic curves of the 1:4 transparent UV PD were measured both in darkness and under illumination and compared with those of the 1:1 counterpart, as demonstrated in Figure 5a and Figure 5b, respectively. The dark I-V curves in Figure 5a vary nonlinearly with the applied voltage, demonstrating the formation of rectifying Schottky junctions at the Au/ZnO interfaces. Meanwhile, they look almost symmetric at positive and negative biases for both devices, meaning the almost equal Schottky barrier heights at the opposite sides. At the zero bias, both dark currents are less than 10^−10^ A, and the dark current of the 1:4 PD is even lower than 10^−11^ A, while at the non-zero biases, the 1:4 PD has dark currents about one order of magnitude lower than the 1:1 PD. In the 1:4 device, the carriers under the wide fingers are not collected as fully as in the 1:1 device with narrow fingers, contributing to the lower dark currents and favorable for low-noise photodetection.

Under UV illumination, the currents increase by several orders of magnitude for the two devices as shown in Figure 5b. The light I-V curve is still almost symmetric for the 1:1 device, but became asymmetric for the 1:4 device. This means the formation of asymmetric Schottky junctions with different barrier heights in the latter device (to be explained in the next section). Based on the dark and light I-V curves (Figure 5a,b), responsivity, R, and specific detectivity, D*, at three low voltages, i.e., 0, 0.5, and 1.0 V, are calculated with the formulas of R = (I_p_ − I_d_)/PS and D* = R/(2eI_d_/S)^1/2^, respectively (where P = 8.62 mW/cm^2^ is the UV light intensity, S = 0.08 cm^2^ is the aIDT area, and e is the electron charge). At 0, 0.5, and 1.0 V, R = 0.0236 μA/W, 1.18, 2.48 mA/W and 0.636 μA/W, 2.41, 4.86 mA/W and the corresponding D* = 2.19 × 10^6^, 4.07 × 10^10^, 7.37 × 10^10^ Jones and 1.05 × 10^8^, 3.41 × 10^11^, 4.15 × 10^11^ Jones for the 1:1 and 1:4 devices, respectively. Through comparison, it is easy to see that the PD with asymmetric IDT electrodes has a much better low-voltage photodetection capability with lower noise and higher specific detectivity. Transient responses of the 1:4 transparent UV PD at 0, 0.5 and 1.0 V were further characterized and plotted in Figure 5c, Figure 5d, and Figure 5e, respectively. Stable and reproducible photocurrents are achieved at different voltages. At zero bias, the rise and fall times of the device are 38.2 and 50.6 ms, respectively (Figure 5c). At 0.5 and 1.0 V, the device becomes much quicker with the rise/fall time reduced to within 10/5 ms. Such fast responses result from not only the small spacing between the aIDT fingers but also the Schottky junction formed at the Au/ZnO interface, which drives photogenerated carriers quickly to the opposite electrodes for collection.

All the characteristic parameters of the 1:4 transparent UV PD are listed in Table 1 and compared with other previously reported low-voltage transparent UV PDs working in the 320–400 nm range [3,4,10,11,14,15,17,18,22]. Due to the highly transparent ultrathin Au film constructed into a simple, in-plane aIDT form, our PD is as transparent as and even more transparent than most reported devices [3,10,11,14,15,18]. Despite the small responsivity, our device still achieves a comparable and even superior specific detectivity at 1.0 V to the previous devices working at the same voltage [4,10,18]. This is mainly due to the very low dark currents, which are at least 3 orders of magnitude smaller than those of most previous devices [3,4,10,14,15,18]. Our PD is also more advantageous in terms of response speed, especially at 0.5 and 1.0 V. The low responsivity is likely due to the poor crystallinity of the sputtered ZnO active layer [23], which may have too many traps for photocarrier recombination. Utilization of a single crystalline ZnO active layer is predicted to significantly improve the responsivity [32]. Overall, our 1:4 transparent UV PD can completely compete with the existing transparent UV PDs in terms of optical transparency and comprehensive performance under low voltages.

### 3.3. Working Mechanism of Transparent UV PD with aIDT Electrodes under Low Voltages

**Figure 6:**
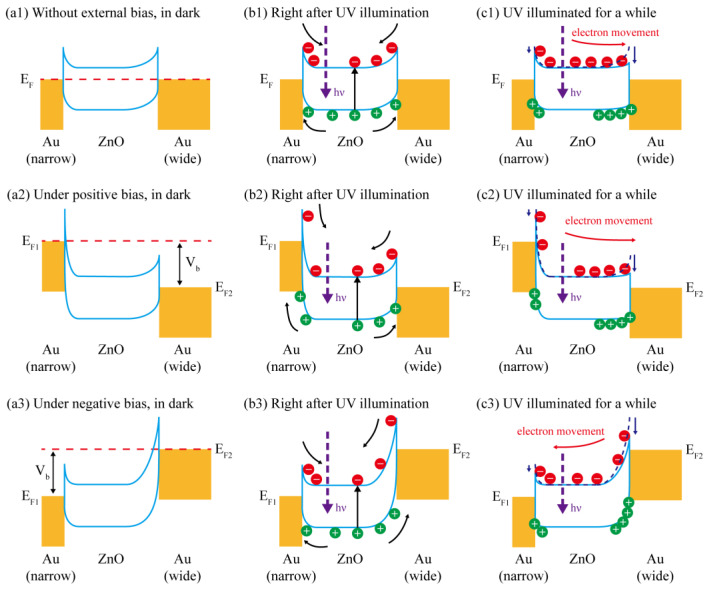
Schematic energy band diagrams of transparent UV PDs with 1:4 aIDT electrodes (**a1**–**a3**) in darkness, (**b1**–**b3**) right after UV illumination and (**c1**–**c3**) being illuminated for a while under (**a1**,**b1**,**c1**) zero, (**a2**,**b2**,**c2**) positive and (**a3**,**b3**,**c3**) negative biases.

As mentioned above, Schottky junction is formed at the Au–ZnO interface, and asymmetric junctions exist at the opposite sides of the device with aIDT electrodes upon UV illumination, enabling high low-voltage performance. For further illustration, schematic energy band diagrams of the unbiased and biased devices in darkness, right after UV illumination and being illuminated for a while are plotted in Figure 6 and analyzed below.

Because of the higher work function of Au than that of ZnO, when Au is deposited on ZnO, both the conduction band and the valence band of the ZnO near the Au–ZnO interface are bent up, where a Schottky barrier is formed with a built-in electric field pointing from ZnO to Au. In the MSM UV PD in darkness, driven by the built-in electric fields with opposite directions at the back-to-back junctions, the electrons in ZnO are blocked to transport to Au while the holes in ZnO are trapped in the depletion regions. The depletion region with the wide Au fingers is wider [33], and therefore, may accommodate more holes than that with the narrow Au fingers. However, the carriers, especially the holes, in ZnO are quite few and the rather small difference in the amount of the trapped holes at the opposite junctions will introduce little influence on the electric potentials. Thus, the Schottky barrier heights depend mainly on the work functions of Au and ZnO as well as their interfacial states [34], and are nearly independent of the size of the Au electrode [26]. In this case, regardless the bias, the junctions with narrow and wide Au fingers have almost the same Schottky barrier height (Figure 6(a1–a3)), leading to a symmetric dark I-V curve for the device with the 1:4 aIDT electrodes similar to that of the 1:1 device (Figure 5a). Because of the electrons blocked and the holes trapped in ZnO, the dark current is quite small, even under biases (Figure 5a and Table 1).

For the unbiased 1:4 device in Figure 6(b1), upon UV illumination, a large number of photoelectrons and photoholes are generated in ZnO and drove to the central ZnO and the back-to-back Au/ZnO interfaces, respectively, under the built-in electric fields in the depletion regions. Eventually, the depletion region under the wide (instead of narrow) Au fingers accommodates much more photoholes. Here, so many photoholes inevitably introduce a larger increment in the electric potential and thus a larger reduction in the effective Schottky barrier height. In this case, imbalanced junctions exist at the opposite sides, i.e., a lower barrier height at the interface with wide fingers and a higher barrier height at the interface with narrow fingers (Figure 6(c1)). Driven by the built-in electric field in the junction with the narrow fingers, photogenerated electrons can easily cross over the lower barrier at the opposite side and be collected by the wide Au finger electrodes, forming a photocurrent much higher than the dark current (Figure 4 and Figure 5a,b). In contrast, for the unbiased 1:1 device with symmetric junctions at the opposite sides, the photoelectrons cannot be effectively extracted and therefore the photocurrent is immeasurable (Figure 4 and Figure 5b). Note that interfacial recombination when the photoelectrons are driven to the wide-finger electrodes is inevitable. Therefore, the photocurrent is actually generated by the photoelectrons unrecombined with the accumulated photoholes at the interface under the wide-finger electrodes.

As shown in Figure 6(a2,b2,c2), under a positive bias of V_b_, the Fermi level of the narrow-finger electrode is raised up. Together with the built-in electric field, the photogenerated electrons are more strongly driven to the opposite side compared to the zero-biased case as shown in Figure 6(a1,b1,c1). Therefore, photocurrent increases with the increasing positive applied voltages. Even a small voltage enables a large photocurrent (Figure 5b). Under a negative bias of V_b_, the Fermi level of the wide-finger electrode is increased (Figure 6(a3,b3,c3)). The photoelectrons are blocked to cross over this lower barrier and driven to the opposite junction with a higher barrier. Since there are still many photoelectrons gaining enough energy to cross over this higher barrier, the photocurrent is much larger than that at 0 V. However, due to the higher barrier for the photoelectrons to cross over, the photocurrent under a negative bias is slightly lower than that under the same positive bias, and the light I-V curve appears to be clearly asymmetric (Figure 5b).

**Figure 7:**
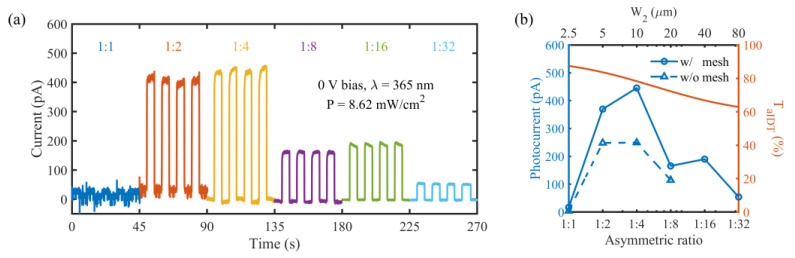
(**a**) Transient output current of the transparent UV PDs with meshed pads and aIDT electrodes of different asymmetric ratios under zero bias when the UV light (*λ* = 365 nm, P = 8.62 mW/cm^2^) is switched on and off periodically. (**b**) Photocurrent extracted from the transient output current and transmittance at 365 nm of the aIDT area, T_aIDT_ (%), as the asymmetric ratio of the aIDT electrodes varies for the transparent UV PDs with meshed pads. Here, photocurrents of the UV PDs with solid pads and W_1_:W_2_ = 1:1, 1:2, 1:4, and 1:8 are also plotted for comparison.

The above working mechanism is further illustrated through studying the effect of the asymmetric ratio of the aIDT electrodes on the optoelectronic conversion performance of the PD under zero bias. Figure 7a shows the transient output currents of the transparent UV PDs with aIDT electrodes of different asymmetric ratios when the illuminated UV light is switched on and off periodically. From these curves, the photocurrent (=I_p_ − I_d_) is extracted for each device and plotted in Figure 7b, where the transmittance at 365 nm of the aIDT electrodes, T_aIDT_, of each device is also plotted. Here, the T_aIDT_ values are calculated with the formula below, which has been verified experimentally in our previous work [35]:

T_aIDT_ = T_film_·f + 100%·(1 − f), (2)

where T_film_ = 57.5% is the transmittance of the 7 nm thick Au film at 365 nm shown in Figure 2b, and f = (W_1_ + W_2_)/(W_1_ + W_2_ + 2·G) is the Au film filling ratio in a pitch, changing with the asymmetric ratio of the aIDT electrodes, i.e., W_1_:W_2_. As defined in Figure 3a, W_1_ = 2.5 μm and G = 6 μm. Therefore, the asymmetry becomes more pronounced with the increasing W_2_, and simultaneously, the shadows of the aIDT electrodes increase, leading to the decrease in UV transmittance at 365 nm or in the absorption of the ZnO active layer of the PD (Figure 7b). In contrast, the photocurrent does not change monotonously with the asymmetric ratio. When W_1_:W_2_ changes from 1:1 to 1:4, the photocurrent increases quickly to the peak. This illustrates that the junction barriers on the two sides differentiate increasingly largely, favorable for the transportation and extraction of photoelectrons. This effect even overcomes the UV transmittance degradation, contributing to the rising photocurrent. Further increasing W_2_ strengthens the asymmetry of the aIDT electrodes and the imbalance of the Schottky junctions. However, the absorption of the ZnO active layer is degraded with the reduced UV transmittance and on the other hand, the interfacial recombination may also increase with the enlarged electrodes. These factors are responsible for the decreasing trend of the photocurrent (Figure 7). The peak photocurrent exists at W_1_:W_2_ = 1:4. For comparison, UV PDs with solid pads and W_1_:W_2_ = 1:1, 1:2, 1:4, and 1:8 were fabricated and the transient currents were measured (Figure S1). The extracted photocurrents were plotted in Figure 7b. Interestingly, similar behaviors are observed for PDs with solid and meshed pads. These facts again confirm the importance of the imbalanced junctions under the aIDT electrodes to the self-powered capability. Compared to the PDs with solid pads, higher photocurrents are observed for the aIDT PDs with meshed pads. This is probably due to the extra photocarriers generated under the meshed pads near the aIDT area. The photoelectrons are driven by the built-in electric field under the Au mesh next to the aIDT area, where the asymmetric junctions quickly sweep them to the opposite finger electrodes, to be collected. Despite the lesser conductivity of the mesh and the inevitable recombination of the photoelectron transport from the meshed pads to the aIDT electrodes, the remaining photoelectrons still contribute to the photocurrent. As the finger width ratio, W_1_:W_2_, varies from 1:1 to 1:4, the asymmetry of the aIDT electrodes becomes more pronounced, and the sweeping effect also becomes stronger, leading to an increasing deviation in the photocurrent between the PDs with meshed and unmeshed pads. Further increasing the asymmetry of the aIDT electrodes introduces greater interfacial recombination under the wide-finger electrodes, compromising the electron-sweeping effect of the asymmetric junctions. Therefore, at W_1_:W_2_ = 1:4, there exists the largest deviation in photocurrent between the PDs with meshed and solid pads. Note that a measurement error, caused by the poor contacts between the Au electrodes and the probes, may happen for the 1:8 meshed device, whose photocurrent is smaller than that of the 1:16 device (Figure 7b).

## 4. Conclusions

In conclusion, we have proposed and experimentally demonstrated a transparent MSM UV PD based on sub-10 nm thick Au aIDT electrodes that exhibits high performance at low voltages. A 7 nm thick Au film, which has a visible transmittance of 80.4% and a R_sh_ of 11.55 Ω/sq, was patterned into aIDT electrodes with different finger widths on the two sides. It is highly transparent, with an *T_avg_* of 74.3% in the aIDT region. The meshed pads further improve the overall transmittance of the device. The 1:4 PD performs the best among all fabricated devices. It has very low dark currents at 0, 0.5, and 1 V, allowing for high responsivities and specific detectivity with UV light. It is also a fast device, especially when working at 0.5 and 1 V. The comprehensive performance is comparable and even superior to those of the reported devices (Table 1). Systematic studies show that the asymmetric Schottky junctions induced by the aIDT electrodes under UV illumination are the main reason for the low-voltage operation of the PD. Additionally, due to the simple and scalable fabrication process, our transparent UV PD has great promise to be applied widely.

## Figures and Tables

**Table 1:** Comprehensive comparison between our 1:4 transparent UV PD and the previously reported low-voltage, transparent UV PDs working in the 320–400 nm range.

PD Architecture	T ^a^	Bias (V)	I_d_ (A)	R (A/W)	D* (Jones)	t_r_/t_f_	Refs.
Ag NWs/TiO_2_/FTO, vertical	70% (device)	0	~1 n	32.5 m	~6 × 10^9^	44 ns/1.85 µs	[3]
NiO/ZnO/ITO, vertical	74.8% (device)	0	1.8 p	20 μ	7.2 × 10^11^	41 µs/71 µs	[11]
Ag NWs/NiO/TiO_2_/FTO, vertical	44.2% (0.4–1 μm; device)	0	~57 μ	136 m	1.11 × 10^9^	1.5 ms/1.7 ms	[14]
Ag NWs/ZnO NWs/Ag NWs, vertical	75% (device)	0.5	53.3 n	4.16	1.59 × 10^13^	1.83 s/1.75 s	[15]
GaN MSM ITO IDT	—	0.5	0.1 n	0.9	—	—	[22]
ZnO/CdO NFs ^b^ MSM Ti/Au w/o IDT	~95% (active layer)	0.5	—	1	—	3 s/3 s	[17]
Ag_x_O/TiO_2_/ITO, vertical	70% (device)	−1	0.65 m	0.323	4.2 × 10^8^	287 ms/361 ms	[10]
TiO_2_ MSM FTO IDT	~80% (device)	1	33.1 n	67.15 m	2.71 × 10^12^	1.71 s/3.17 s	[4]
ZnO:Ag NWs MSM Au w/o IDT	75% (active layer)	1	15.0 n	2.4	6.8 × 10^12^	3.53 s/3.67 s	[18]
ZnO MSM 7 nm Au aIDT	74.3% (weighted with V(*λ*); device)	0	9.18 p	0.636 μ	1.05 × 10^8^	38.2 ms/50.6 ms	This work
		0.5	12.5 p	2.41 m	3.41 × 10^11^	8.55 ms/3.86 ms	
		1	34.1 p	4.86 m	4.15 × 10^11^	9.16 ms/4.82 ms	

^a^ T, averaged transparency values in the visible regime and without weighting calculation unless otherwise specified. ^b^ NFs, nanofibers.

## Data Availability

Data underlying the results presented in this paper are not publicly available at this time but may be obtained from the authors upon reasonable request.

## Supplementary Materials

The following supporting information can be downloaded at: https://www.mdpi.com/article/10.3390/mi14071447/s1. Figure S1: Transient output current of the non-meshed transparent UV PDs with aIDT electrodes of different asymmetric ratios under zero bias when the UV light (*λ* = 365 nm, P = 8.62 mW/cm^2^) is switched on and off periodically.
