# Supplementary material for: High-Performance Low-Voltage Transparent Metal-Semiconductor-Metal Ultraviolet Photodetectors Based on Ultrathin Gold Asymmetric Interdigitated Electrodes
Source: Micromachines (Basel). 2023 Jul 19;14(7):1447. doi: 10.3390/mi14071447 (PMC10386012; doi:10.3390/mi14071447)
Supplement: Supplementary file 1 [file micromachines-14-01447-s001.zip › micromachines-2489607-supplementary.pdf]

micromachines

Supplementary Information: High-Performance Low-Voltage Transparent Metal-Semiconductor-Metal Ultraviolet Photodetectors Based on Ultrathin Gold Asymmetric Interdigitated Electrodes

Jianfeng Huang [1], Liu Yang [1,2,*] and Sailing He [1,2,3,*]

1 Centre for Optical and Electromagnetic Research, National Engineering Research Center for Optical Instruments, College of Optical Science and Engineering, Zhejiang University, 866 Yuhangtang Road, Hangzhou 310058, China
2 Ningbo Research Institute, Zhejiang University, Ningbo 315100, China
3 Joint Research Center of Photonics, School of Electrical Engineering, Royal Institute of Technology (KTH), S-100 44 Stockholm, Sweden
* Correspondence: optyang@zju.edu.cn (L.Y.); sailing@zju.edu.cn (S.H.)

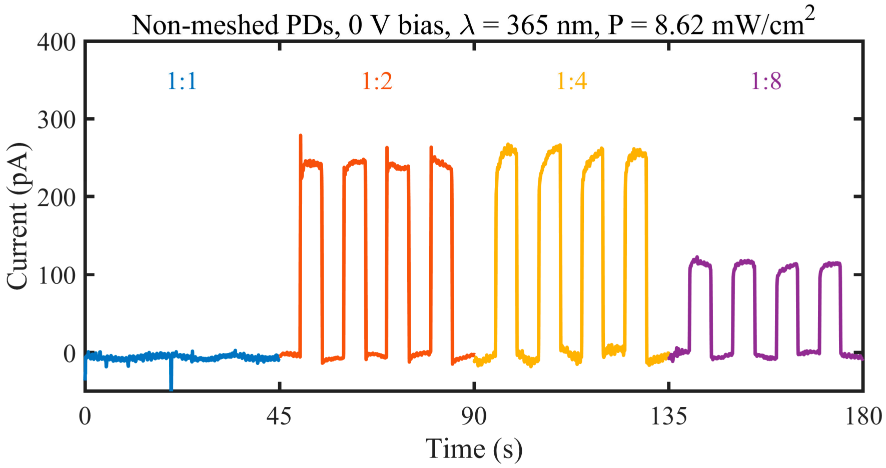

Figure S1. Transient output current of transparent UV PDs with non-meshed pads and aIDT electrodes of different asymmetric ratios under zero bias when the UV light (λ = 365 nm, P = 8.62 mW/cm^2) is switched on and off periodically.
